# The First Two *Clostridium difficile* Ribotype 027/ST1 Isolates Identified in Beijing, China–an Emerging Problem or a Neglected Threat?

**DOI:** 10.1038/srep18834

**Published:** 2016-01-07

**Authors:** Jing-Wei Cheng, Meng Xiao, Timothy Kudinha, Zhi-Peng Xu, Xin Hou, Lin-Ying Sun, Li Zhang, Xin Fan, Fanrong Kong, Ying-Chun Xu

**Affiliations:** 1 Department of Clinical Laboratory, Peking Union Medical College Hospital, Chinese Academy of Medical Sciences, Beijing 100730, China; 2 Graduate School, Peking Union Medical College, Chinese Academy of Medical Sciences, Beijing 100730, China; 3 Charles Sturt University, Leeds Parade, Orange, New South Wales 2687, Australia; 4 Centre for Infectious Diseases and Microbiology Laboratory Services, ICPMR – Pathology West, Westmead Hospital, University of Sydney, Darcy Road, Westmead, New South Wales 2145, Australia; 5 Teaching and Research Section of Clinical Laboratory, School of Public Health, Taishan Medical School, Taian, Shandong 271000, China

## Abstract

*Clostridium difficile* hyper-virulent ribotype 027 strain has become a significant concern globally, but has rarely been reported in Asian countries including China. Recently, a retrospective single-center study in Beijing, China, detected two ribotype 027 *C. difficile* isolates from two patients coming for outpatient visits in 2012 and 2013. We performed a systematic investigation of the two isolates (and patients). Both *C. difficile* isolates had the typical PCR ribotype 027 profile; were positive for *tcdA*, *tcdB* and binary toxin genes; belonged to multilocus sequence type 1 (ST1); had typical ribotype 027 deletions in the *tcdC* gene; and were highly-resistant to fluoroquinolones; but had a different MLVA profile and were not genetically related to any previously reported international ribotype 027 clones. A review of the patients’ medical records showed that neither received appropriate antimicrobial treatment and were lost to follow-up after outpatient visits. We propose that *C. difficile* infections caused by ribotype 027 are probably a neglected problem in China, and the subsequent impact of unawareness of this problem is worrying. Appropriate testing assays and multi-center or national level surveillance for *C. difficile* infections and specifically for ribotype 027 should be introduced to provide essential data and guide future clinical practice.

*Clostridium difficile* is a Gram-positive, spore-forming, anaerobic rod. *C. difficile* infection (CDI) is a major nosocomial disease which is associated with high morbidity and potential mortality^1^,^2^. Since the 2000s, there has been a significant increase in the incidence and severity of CDI in North America and Europe, placing a huge financial burden on health care systems worldwide^1^,^3^. The emergence of the hyper-virulent *C. difficile* strain BI/NAP1/027 (restriction endonuclease analysis group BI, North American pulse-field type 1, PCR ribotype 027) has substantially contributed to the rise in CDI incidence^4^. The hypervirulence of the epidemic strains is likely due to the polymorphisms in the *tcdB* receptor-binding domain, leading to a hypertoxic and antigenically variable form of TcdB^5^. In addition, this strain possesses an extra toxin known as binary toxin, which is associated with increased clinical severity. A further characteristic of *C. difficile* ribotype 027 is an increased *in vitro* sporulation rate in the absence or presence of non-chloride cleaning agents^6^. In contrast to historic strains of ribotype 027 *C. difficile*, the new epidemic strains are resistant to fluoroquinolones^4^,^7^. After the detection of the first case of ribotype 027 in North America, this epidemic strain has subsequently spread rapidly in Europe and North America^8^.

However, CDI is not widely recognized in Asia including China, possibly due to insufficient laboratory diagnostic capacity, low sample submission rate, and lack of high-quality surveillance systems^8^,^9^. Compared to North America and Europe, relatively few studies on *C. difficile* have been performed in Asia, with *C. difficile* ribotype 027 occasionally reported in the region to date. Thus the genuine extent of the disease and burden of *C. difficile* ribotype 027 in China and the majority of Asian countries remains largely unknown. Based on this background, and in order to gain some insights on CDI epidemiology in China, we retrospectively tested stool specimens (from suspected CDI cases), from a major hospital in Beijing (Peking Union Medical College Hospital, PUMCH), using culture and molecular tests.

In this retrospective study, two *C. difficile* ribotype 027 isolates were detected, which are the first reported cases in Beijing, China. Furthermore, we characterized isolates by molecular approaches and antimicrobial susceptibility testing and reviewed patients’ medical records, aiming to have a better understanding of the current situation for future clinical practice implications.

## Results

### Background of *C. difficile* isolates

Out of 336 stool specimens from suspected CDI cases, 94 (28.0%) were positive by culture, but only 36 (10.7%) were positive for toxin A and/or B, by enzyme immunoassay (EIA). Further toxin testing on the *C. difficile* isolates yielded four isolates positive for toxin A and B genes (*tcdA*, *tcdB*) and the binary toxin genes (*cdtA* and *cdtB*).

### Confirmation of two *C. difficile* ribotype 027 isolates

By capillary sequencer-based PCR ribotyping and querying against WEBRIBO database, two of the four above-mentioned binary toxin gene positive *C. difficile* isolates, namely PUCD235 and PUCD301, were confirmed as ribotype 027. Both isolates produced eight peaks, which were of identical pattern to that produced by previous well-characterized PCR ribotype 027 strain CA2^10^,^11^.

### Genotypes

By MLST, the 94 *C. difficile* isolates were classified into 22 STs, and the majority (58.5%) belonged to clade 1. ST-3 was the most common ST (18.1%, 17/94), followed by ST-54 (16.0%, 15/94), ST-37 (10.6%, 10/94) and ST-81 (9.6%, 9/94). The two ribotype 027 isolates (PUCD235 and PUCD301) belonged to ST1. In addition, sequencing of the *slpA* gene of the two isolates revealed a single nucleotide polymorphism (SNP), C467T, which was different from most previous *C. difficile* ribotype 027 isolates world-wide (*slpA* sequence type gc-8, e.g. strains CD196, BI1, QCD-66c26, and CIP 107932, genome accession nos. FN538970, FN668941, CM000441 and CM000659, respectively)^12^,^13^, with an amino acid substitution from proline to leucine at residue 156, but was identical to the *slpA* gene sequence of strain 2007855 (genome accession no. FN665654) from USA^12^.

### Multiple-locus variable-number tandem-repeat analysis (MLVA)

**Figure 1:**
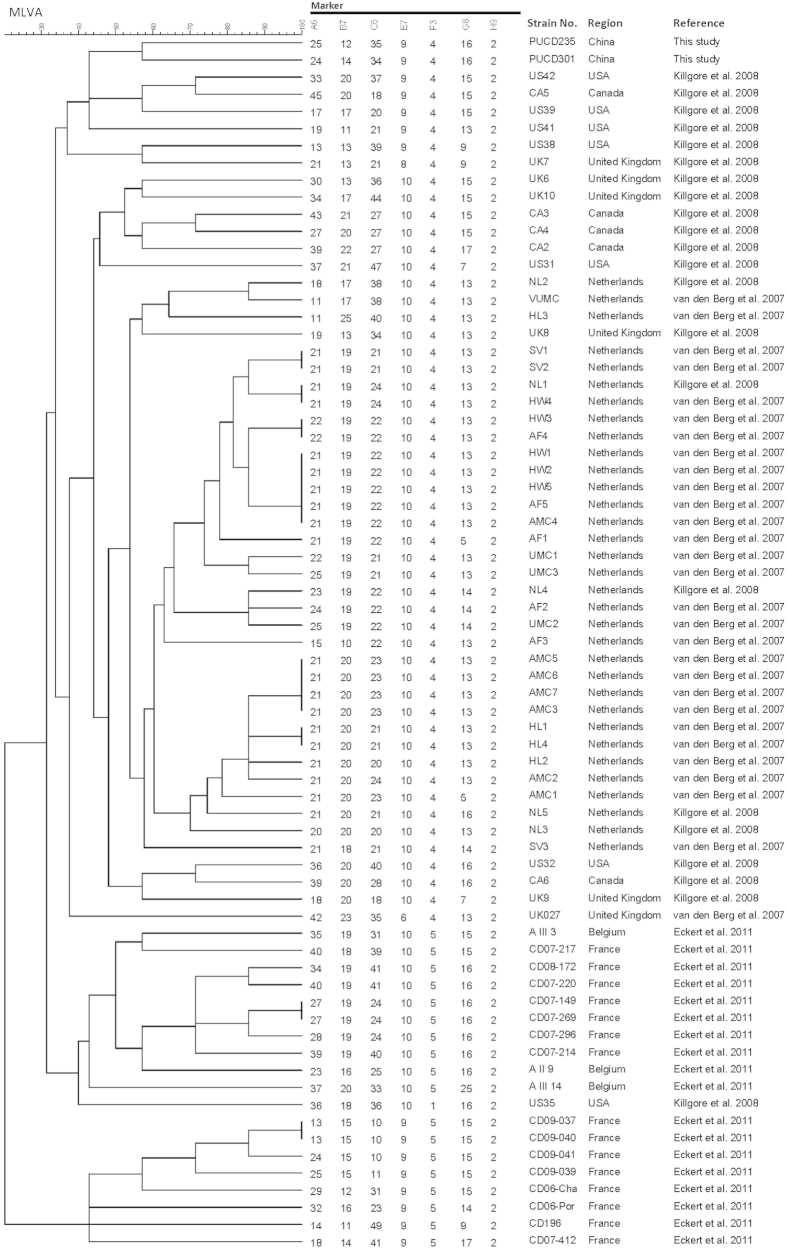
MLVA dendrogram based on profiles of seven markers for the two ribotype 027 isolates (n = 2) in this study and other previous reported strains (n = 69) 10,14,15.

MLVA was performed to investigate the genetic relatedness among Chinese, European and North American *C. difficile* ribotype 027 isolates. In three previous studies, 69 ribotype 027 *C. difficile* isolates from the USA, Canada, United Kingdom, France and Netherlands, were investigated by MLVA^10^,^14^,^15^. Regarding the two ribotype 027 isolates, they differed by 3 markers (A6_*Cd*_, B7_*Cd*_ and C6_*Cd*_), and had a similarity of about 57%. As shown in Fig. 1, the two ribotype 027 isolates were also not genetically related with other previously reported ribotype 027 strains (Fig. 1).

### Characterization of toxin genes

Of the 94 *C. difficile* isolates studied, 51 (54.3%) were *tcdA* and *tcdB*-positive, and *cdtA/cdtB*-negative (A^+^B^+^CDT^−^), whilst 20 (21.3%) were *tcdA*-negative, *tcdB*-negative and *cdtA/cdtB*-negative (A^−^B^−^CDT^−^), and 19 (20.2%) were *tcdA*-negative, *tcdB*-positive and *cdtA/cdtB*-negative (A^−^B^+^CDT^−^). The remaining four (4.2%) isolates were *tcdA*-positive, *tcdB*-positive and *cdtA/cdtB*-positive (A^+^B^+^CDT^+^). The *tcdC* gene sequences of the two ribotype 027 isolates revealed a single-nucleotide deletion at position 117 and an 18-bp deletion at position 330–347, resulting in an inactivating frame-shift mutation in the *tcdC* gene of these isolates when compared with the sequence of wild-type *tcdC* gene (*C. difficile* 630, genome accession no. NC_009089), which was identical to previous published ribotype 027 genome sequences^13^,^16^.

### Antimicrobial susceptibilities

The two *C. difficile* PCR ribotype 027 isolates had similar antimicrobial susceptibilities, being resistant to clindamycin, erythromycin, fluoroquinolones (ciprofloxacin and levofloxacin), rifampicin, rifaximin, but susceptible to metronidazole, meropenem, piperacillin/tazobactam, tetracycline and vancomycin (Table 1).

### Medical records review

The two ribotype 027 isolates were from two individual patients who visited an outpatient clinic at Peking Union Medical College Hospital. The first isolate, PUCD235, was from a 58-year-old male patient who came to the outpatient clinic on December 11, 2012 post rectal cancer surgery at another hospital. The second isolate, strain PUCD301, was from a 59-year-old female patient who visited the hospital for cholecystitis on March 17, 2013. Both patients had symptoms of diarrhea upon visiting, and increased white blood cell counts and neutrophils were noticed. However, laboratory routine testing for *C. difficile* toxins A and B by the commercial EIA methods for both patients were negative, and no additional tests (e.g. *C. difficile* culture) were ordered by the clinicians. Consequently, CDI was not diagnosed, and thus the patients did not receive appropriate antimicrobial treatment and were subsequently lost to follow-up.

### *C. difficile* ribotype 027 reported in Asian countries

**Figure 2:**
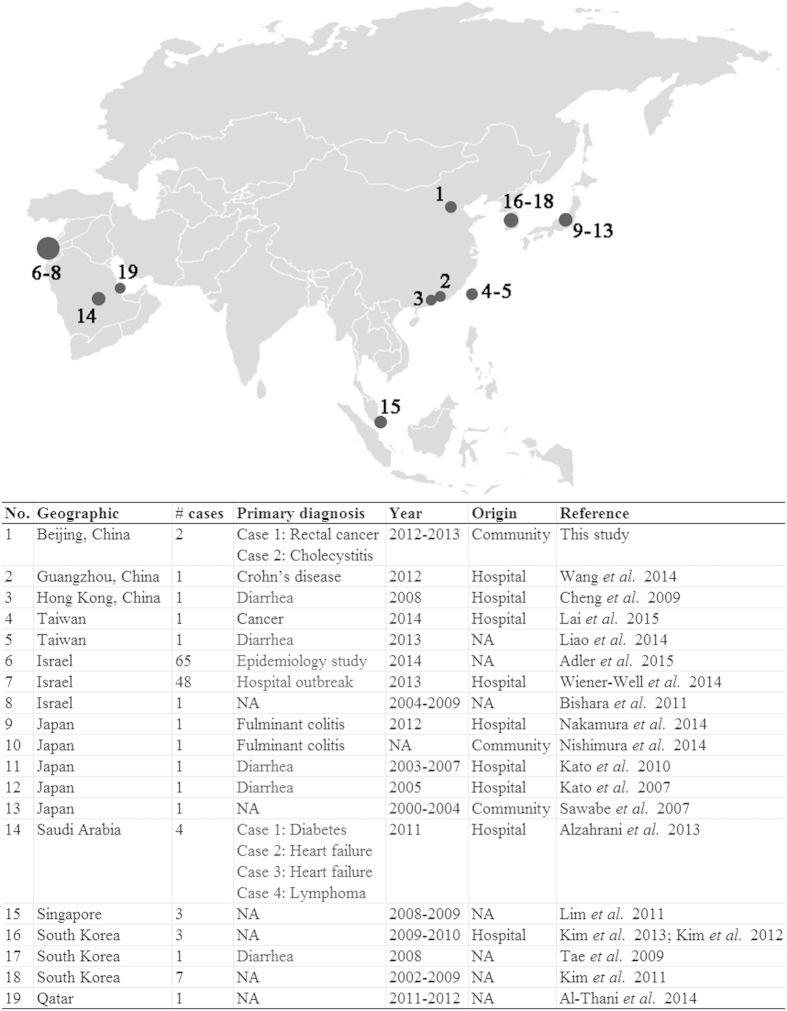
Summary of *C. difficile* PCR ribotype 027 reported in Asia. Size of circles indicated the number of cases identified in each region. NA, data not available. The map was generated by GNU Image Manipulation Program (version 2.8.14, the GIMP Team, USA).

By searching on PubMed (http://www.ncbi.nlm.nih.gov/pubmed) using “*Clostridium difficile”* and “Asia” as the keywords, a total of 206 articles were found as of November 6, 2015. Among these, 19 full text articles reporting on the detection of *C. difficile* ribotype 027 isolates were found, with 142 cases identified in eight regions (Fig. 2)^17^,^18^,^19^,^20^,^21^,^22^,^23^,^24^,^25^,^26^,^27^,^28^,^29^,^30^,^31^,^32^,^33^,^34^,^35^. In 2000, the first *C. difficile* ribotype 027 case was reported in Japan by Sawabe *et al.*^29^. To date, most of the *C. difficile* ribotype 027 isolates identified in Asia (114/142, 80.3%) were reported in Israel^17^,^20^,^31^, including 65 isolates from a local national epidemiology study^17^, and 48 isolates from an outbreak^31^. In addition, 11 (7.7%) isolates were reported in South Korea^23^,^24^,^30^,^34^. The remaining 17 *C. difficile* ribotype 027 isolates were sporadically reported case-by-case from Japan (n = 5)^22^,^28^,^29^,^32^,^33^, Saudi Arabia (n = 4)^19^, Singapore (n = 3)^27^, mainland China (n = 1)^35^, Taiwan (n = 2)^25^,^26^, Hong Kong (n = 1)^21^, and Qatar (n = 1)^18^ (Fig. 2).

## Discussion

CDI is a major medical and infection control threat to health care facilities, including hospitals, long-term care facilities, and nursing homes around the world. The majority of CDI cases reported recently are largely associated with the emergence of epidemic ribotype 027 strain, with most of it reported from North America and Europe^36^,^37^,^38^. However, according to our literature review, only 142 ribotype 027 cases were reported in Asia from 19 publications in eight regions, with 80.3% of these cases reported from Israel, where ribotype 027 has become the major clone disseminated in that country^17^,^20^,^31^. For most Asian countries, *C. difficile* ribotype 027 cases have only been reported sporadically.

The few reported cases of *C. difficile* ribotype 027 in Asia may be a tip of the iceberg as many Asian countries have inadequate laboratory diagnostic capacity, low submission rate of samples, and lack high-quality and multiple-center surveillance systems for CDI. According to our previous survey data, as of Dec 2014, only 70 amongst over 1700 tertiary hospitals in China, routinely carried out EIA for detection of *C. difficile* toxins A and B (unpublished data), which was the only commercial *C. difficile* testing method approved by China Food and Drug Administration then. Furthermore, few laboratories perform culture or molecular-based detection of *C. difficile*. Therefore, the magnitude of the CDI problem may be much under-estimated^8^,^9^.

To date, only one CDI case due to *C. difficile* ribotype 027 has been reported in mainland China, so the two cases identified in the present study are the second and third reported cases in China. Of note, the two patients involved did not get accurate diagnosis of CDI upon outpatient visit, mainly because the EIA testing for *C. difficile* toxins A and B were negative, although both patients had symptoms of diarrhea and abnormal routine blood test results. It has been reported that EIA-based methods are comparably less sensitive and have lower positive predictive value than toxigenic culture or molecular tests, in detecting *C. difficile* toxins. Moreover, the VIDAS EIA assay also appears to have lower sensitivity than other EIA-based methods for the detection of CDI^39^, prompting many centers to implement a two-pronged strategy that combines glutamate dehydrogenase (GDH) assay and toxin detection results to diagnose CDI. The two patients did not receive any appropriate treatment and were lost to follow-up which is a concern as these un-recognized *C. difficile* cases may become important transmission sources and a potential threat to public health^40^. Our findings suggest that *C. difficile* ribotype 027 has existed in northern China, therefore, it is important to improve laboratory diagnostic capacity and carry out active surveillance for the emergence of *C. difficile* hyper-virulent clones to avoid potential epidemic spread.

The development of molecular typing methods is of great significance for better understanding of *C. difficile* epidemiology. Our study identified the similarities and differences between the present *C. difficile* ribotype 027 isolates and globally described epidemic strains. Specifically, if only looking at MLST data, both strains belonged to ST1, suggesting possible similar origin source. However, a number of the 027 isolates previously reported in Asia were from a “historic” strain, which was fluoroquinolone susceptible, which is in contrast to the strains described in this study that were clearly fluoroquinolone resistant. In addition, sequencing of the *slpA* gene revealed that the two strains shared a SNP C467T, which differed from most global ribotype 027 strains, but was similar to one USA strain (2007855) recovered from animal rather than human source^12^. Further MLVA typing results also supported that the two ribotype 027 isolates were genetically different as they did not cluster together with strains from previously reported cases in North America and European countries (Fig. 1). The high discriminatory power of MLVA typing, if used properly (with suggested reasonable cut-off value) can be a potential valuable tool for investigating future ribotype 027 outbreaks^41^. In addition, if an association can be established between specific ribotype 027 MLVA subtypes, and virulence and clinical outcome of CDI patients^42^, this would be valuable for infection control and may help explain why our two strains appeared to have low virulence.

In conclusion, our study characterized the first two *C. difficile* PCR ribotype 027 isolates identified in Beijing, China. Our findings highlight the importance of raising public awareness, improving the laboratory diagnostic capacity, as well as implementing active surveillance systems for CDI in China, so as to capture this neglected potential public health threat and for providing useful indications for future clinical and infection control practices.

## Methods

### Ethics

The study was approved by the Human Research Ethics Committee of Peking Union Medical College Hospital (No. S-263), and the study was carried out in accordance with the approved guidelines. Informed consents were obtained from all the patients.

### Background for local laboratory diagnosis of *C. difficile* infections

Stool specimens from suspected CDI cases are routinely sent to the PUMCH laboratory in Beijing for *C. difficile* detection. The available tests for *C. difficile* detection at this hospital are toxin A and B detection using a commercial enzyme immunoassay (EIA) (VIDAS *C. difficile* Toxin A&B, bioMerieux, Marcy l’Etiole, France). However, *C. difficile* culture and molecular testing are rarely performed in most Chinese hospitals.

### Retrospective analysis of faecal specimens for *C. difficile*, including culture and molecular assays

A total of 336 consecutive, non-repetitive faecal specimens from 336 patients (stored at −80 °C before use) were collected between August 2012 and July 2014. All the specimens were tested by culture and molecular assays. Generally, the specimens were cultured on cycloserine-cefoxitin fructose agar (CCFA) in anaerobic condition at 35 °C for 48 h, and suspected colonies were identified by matrix-assisted laser desorption/ionization time-of-flight mass spectrometry. Presence of toxin-encoding genes was then detected by a multiplex PCR assay to all confirmed *C. difficile* isolates as described below.

### DNA extraction and toxin gene detection

One to five typical colonies were picked up from pure cultures of *C. difficile* isolates, and bacterial suspensions equivalent to 1 McFarland turbidity standard in 200μl double-distilled water, were made. Genomic DNA was extracted with QIAamp DNA Mini Kit (QIAGEN, Hilden, Germany) according to the manufacturer’s instructions. A 5-plex PCR was used to detect *tcdA*, *tcdB*, *cdtA* and *cdtB* genes and the 16S rDNA, as previously described by Persson *et al.*^43^. We also sequenced the *tcdC* gene, a negative regulator of *tcdA* and *tcdB*^43^.

### Capillary sequencing-based PCR ribotyping

Capillary sequencer-based PCR ribotyping was performed as described by Indra *et al.*^44^. Generally, primers 16S-F (5′-GTGCGGCTGGATCACCTCCT-3′) and 23S-R (5′-CCCTGCACCCTTAATAACTTGACC-3′) were used for amplification, with 16S primer labeled at the 5′ end with carboxyfluorescein (FAM). The PCR product was diluted 1:10 in water before loading. Fragment separation was performed on an ABI 3730 sequencer (Applied Biosystems, Carlsbad, USA) with a 50 cm POP 7 gel. Sample injection was at 1.6 kV over 15 seconds, with a total running time of 6200 seconds. A GeneScan™ 1200 LIZ (Applied Biosystems) size standard was used as internal marker. The results were analyzed by GeneMarker software (Version2.2.0, SoftGenetics, State College, PA, USA), and queried against WEBRIBO database (https://webribo.ages.at/) for ribotypes. A previously well-characterized PCR ribotype 027 isolate, strain CA2, was used as internal control for PCR ribotyping^10^.

### Multilocus sequence typing (MLST)

MLST was performed by sequencing seven gene loci (*adk, atpA, dxr, glyA, recA, sodA* and *tpi*) as previously described by Griffiths *et al.*^45^. DNA sequences were submitted to the PubMLST sequence query page (http://pubmlst.org/cdifficile/) to obtain the sequence type (ST) and clade.

### Sequencing of the *slpA* gene

As described previously by Kato *et al.*^46^, the partial *slpA* gene was amplified by the primers slpAcom19 (5′-GTTGGGAGGAATTTAAGRAATG-3′) and slpAcom22 (5′-GCWGTYTCTATTCTATCDTYWCC-3′). Both strands of the amplified products were sequenced and comparisons were made with the amino acid sequences deduced from the DNA sequences.

### Multilocus variable-number tandem repeat analysis (MLVA)

The genetic relatedness of the toxigenic isolates was investigated by MLVA, using the set of 7 loci (A6_*Cd*_, B7_*Cd*_, C6_*Cd*_, E7_*Cd*_, F3_*Cd*_, G8_*Cd*_, and H9_*Cd*_) as previously described by van den Berg *et al.*^15^. Repeat numbers were analyzed using BioNumerics software v6.5 (Applied Maths, Texas, USA) for cluster analysis. A dendrogram was constructed using the unweighted-pair group method with arithmetic mean clustering (UPGMA) with the multistate categorical similarity coefficient (MCSC).

### Antimicrobial susceptibility testing

Antimicrobial susceptibility testing was performed by the agar dilution method according to Clinical and Laboratory Standards Institute (CLSI) guidelines (document M11-A8)^47^. The following 11 antimicrobial agents were chosen: ciprofloxacin, clindamycin, erythromycin, levofloxacin, meropenem, metronidazole, piperacillin/tazobactam, rifampicin, rifaximin, tetracycline and vancomycin. Interpretation of testing results was based on CLSI M100-S25^48^, or according to the criteria suggested by Huang *et al.*^49^ for the drugs whose breakpoints were not available in CLSI documents, as summarized in Table 1.

### Review of *C. difficile* ribotype 027 reported in Asian countries

To have a more comprehensive understanding of current epidemiology of *C. difficile* ribotype 027 in Asia, we reviewed and summarized all related published literature in PubMed (http://www.ncbi.nlm.nih.gov/pubmed) database as of November 6, 2015.

## Additional Information

**How to cite this article**: Cheng, J.-W. *et al.* The First Two *Clostridium difficile* Ribotype 027/ST1 Isolates Identified in Beijing, China–an Emerging Problem or a Neglected Threat? *Sci. Rep.* **6**, 18834; doi: 10.1038/srep18834 (2016).

## Figures and Tables

**Table 1:** Antimicrobial susceptibility of the two *C. difficile* ribotype 027 isolates identified in this study.

Antimicrobial agents	Resistant breakpoint (μg/mL)	MIC (μg/mL)/category	
		PUCD235	PUCD301
Erythromycin	≥8^b^	≥256/R	≥256/R
Ciprofloxacin	≥8^b^	128/R	64/R
Clindamycin	≥8^a^	256/R	128/R
Levofloxacin	≥8^b^	256/R	128/R
Meropenem	≥16^a^	2/S	2/S
Metronidazole	≥32^a^	1/S	1/S
Piperacillin/tazobactam	≥128/4^a^	4/4/S	4/4/S
Rifampicin	≥4^b^	≥256/R	≥256/R
Rifaximin	≥16^b^	≥256/R	≥256/R
Tetracycline	≥16^a^	0.125/S	≤0.064/S
Vancomycin	≥32^b^	2/S	2/S

Abbreviations: MIC, minimum inhibitory concentration; S: susceptible; R: resistant.

^a^Breakpoints per CLSI document M100-S25.

^b^Breakpoints per Huang *et al.*^49^.
